# First‐line antibiotic treatment in patients with localized extragastric mucosa‐associated lymphoid tissue lymphoma

**DOI:** 10.1002/jha2.608

**Published:** 2022-11-06

**Authors:** Ming Yao, Shu‐Lang Liao, Chung‐Wu Lin, Cheng‐Ping Wang, Wei‐Li Ma, Yi‐Hsuan Wei, Jyh‐Ming Liou, I‐Jong Wang, Ann‐Lii Cheng, Sung‐Hsin Kuo

**Affiliations:** ^1^ Department of Internal Medicine National Taiwan University Hospital and National Taiwan University College of Medicine Taipei Taiwan; ^2^ Department of Ophthalmology National Taiwan University Hospital and National Taiwan University College of Medicine Taipei Taiwan; ^3^ Department of Pathology National Taiwan University Hospital and National Taiwan University College of Medicine Taipei Taiwan; ^4^ Department of Otolaryngology National Taiwan University Hospital and National Taiwan University College of Medicine Taipei Taiwan; ^5^ Department of Oncology National Taiwan University Hospital and National Taiwan University College of Medicine Taipei Taiwan; ^6^ Cancer Research Center National Taiwan University College of Medicine Taipei Taiwan; ^7^ Graduate Institute of Oncology National Taiwan University College of Medicine Taipei Taiwan; ^8^ Department of Internal Medicine National Taiwan University Cancer Center National Taiwan University College of Medicine Taipei Taiwan; ^9^ Department of Medical Oncology, National Taiwan University Cancer Center National Taiwan University College of Medicine Taipei Taiwan; ^10^ Department of Radiation Oncology, National Taiwan University Cancer Center National Taiwan University College of Medicine Taipei Taiwan

**Keywords:** antibiotics, B‐cell lymphoma, extragastric area, *Helicobacter pylori*, mucosa‐associated lymphoid tissue

## Abstract

Between January 2010 and December 2015, we enrolled 28 patients with stage IEI/IIE1 extragastric mucosa‐associated lymphoid tissue (MALT) lymphoma who received first‐line antibiotic treatment, after informing them about the pros and cons of alternative therapies. In addition, during the same period, 64 patients with stage IE/IIE1 disease who received conventional treatment were selected as the control group. The most common primary sites were the ocular adnexal area (17 cases), followed by the salivary glands (four cases), pulmonary (three cases), and thyroid, trachea, larynx, and colon region (one case each). First‐line antibiotic treatment resulted in an overall response rate of 57.1%: 12 patients achieved complete remission (CR), while four achieved partial remission (antibiotic‐responsive tumors). Monoclonal gammopathy was significantly prevalent in antibiotic‐unresponsive tumors than in antibiotic‐responsive tumors (50.0% [6/12] vs. 12.5% [2/16], *p* = 0.044). After a median follow‐up of 7 years, all patients with CR remained lymphoma‐free, with 7‐year event‐free survival (EFS) and overall survival (OS) rates of 62.7% and 96.4%, respectively. The 7‐year EFS and OS rates of patients who received conventional treatments were 73.1% and 91.1%, respectively. Compared with that noted in patients who received conventional treatment, antibiotic treatment was effective in some patients with localized extragastric MALT lymphoma.

## 1 INTRODUCTION

Gastric mucosa‐associated lymphoid tissue (MALT) lymphoma, the most common extranodal marginal zone B‐cell lymphoma, is characterized by *Helicobacter pylori* (HP) infection and is managed with first‐line antibiotic treatment [1, 2]. Previous reports have detected *Chlamydia psittaci* (CP) infection in ocular adnexal MALT lymphoma (OAML) samples and *Borrelia burgdorferi* infection in a proportion of cutaneous MALT lymphomas [3, 4, 5, 6]; however, the association between bacterial infection and other extragastric MALT lymphomas remains unclear. Previous prospective and retrospective studies revealed that approximately 48% of patients with OAML achieved complete remission (CR) and remained lymphoma‐free after the first‐line antibiotic treatment comprising doxycycline to eradicate CP [3, 4, 6, 7].

However, Grünberger et al. showed that none of the 11 patients with OAML in their study responded to first‐line antibiotic treatment with doxycycline, indicating that CP infection may not always be associated with lymphomagenesis in OAML [8]. The discrepancy in doxycycline efficacy in OAML may result from the diverse detection rates of CP DNA in different geographic areas; for example, higher CP infection rates were reported in Western Europe (Italy, Germany, and Austria) and Korea, while infection rates were low in northern China, Japan, and the northeastern United States [9].

Although the use of first‐line antibiotics for the treatment of localized extragastric MALT lymphoma remains unclear, a previous case series revealed that patients with pulmonary MALT, salivary gland MALT, thyroid MALT, or bladder MALT lymphoma accompanied by HP‐positive gastritis achieved CR after receiving first‐line HP eradication (HPE) regimens, such as proton pump inhibitors (PPI) plus clarithromycin, amoxicillin, and metronidazole [10, 11, 12, 13, 14, 15]. These findings indicate that HPE regimens may eradicate antigen stimulation by other bacteria or cause antitumor effects, as clarithromycin exerts antineoplastic effects by triggering apoptosis, and shows immune modulation effects by increasing the cluster of differentiation 8 (CD8) cytotoxicity and natural killer cell function [16, 17]. In a pilot study addressing the efficacy of the long‐term administration of 500 mg clarithromycin twice daily for 6 months for relapsed and refractory MALT lymphoma, Govi et al. reported that patients had an overall response rate (ORR) of 38.5% (10 of 26 patients), including eight patients with CR and two with partial remission (PR) [18].

In summary, these findings indicate that certain patients with localized extragastric MALT lymphoma may benefit from first‐line antibiotic treatment and avoid radiotherapy or chemotherapy‐related adverse effects. In the current study, we assessed whether patients with localized extragastric MALT lymphoma could be cured by first‐line antibiotic treatment, comprising doxycycline, clarithromycin, and HPE therapy regimens. In addition, we selected patients with localized OAML, pulmonary MALT lymphoma, and salivary gland MALT lymphoma who received standard therapeutics, including radiotherapy, chemotherapy, and rituximab‐based regimens, as the control group.

## 2 METHODS

### 2.1 Patients, histologic diagnosis, and study design

Localized extragastric MALT lymphoma is relatively indolent, and several anecdotal case reports have shown that clarithromycin‐based HPE treatment could result in CR in selected patients with extragastric MALT lymphoma, including salivary gland, thyroid, pulmonary, and bladder MALT lymphoma [10, 11, 12, 13, 14, 15]. Therefore, first‐line antibiotics, including doxycycline, clarithromycin, or HP eradication regimens, have also been prescribed for patients with localized extragastric MALT lymphoma and underwent a brief trial of antibiotics after being fully informed about the pros and cons of antibiotic therapy and conventional treatments, including radiotherapy, chemotherapy, or rituximab‐based regimens between January 2010 and December 2015 in our Institute. Patients who want to receive first‐line antibiotic treatment after being well‐informed of the pros (2 weeks of antibiotic treatment may replace at least 3 weeks of radiotherapy or at least four cycles of systemic treatment and further avoid adverse effects) or cons (possible delayed tumor response to conventional treatment) have been protected from non‐supervised lymphoma progression by relatively standard physical examinations and imaging assessments, including endoscopy (colonfibroscopy or bronchoscopy), computed tomography (CT) scan, or magnetic resonance imaging (MRI). If patients did not respond to first‐line antibiotic treatment, they were treated with radiotherapy, chemotherapy, or a rituximab‐based regimen.

To be included in the study, patients had to be diagnosed with localized stage (stage IE and IIE1) and histologically confirmed marginal zone B‐cell lymphoma involving the extranodal side of the extragastric area. Patients with disease evaluable on physical examination, or by endoscopy, CT, or MRI were included in this study. Certain patients with a documented report stating the presence or absence of HP infection before treatment, which was evaluated by the following tests: histology, rapid urease test (Campylobacter‐like organism [CLO]‐test; optional), C^13^ urease breath test, and serology [19], were included in the study. Patients with a history of extranodal lymphoma or stage IIE2, stage III, and stage IV were not eligible. Patients with prior chemotherapy or radiotherapy for extragastric lymphoma were also excluded. If the pathological investigations showed high‐grade transformation, focal large lymphoid cells were excluded from this explorative study.

The diagnosis of MALT lymphoma was based on the World Health Organization (WHO) classification of marginal zone B‐cell lymphoma, in which histological manifestations are characterized by small centrocyte‐like cells, monocytoid B cells, small lymphocytes, and plasma cells with and without lymphoepithelial lesions [4, 20]. Specimens of extranodal MALT lymphoma were immunohistochemically stained for CD20, CD5, CD3, and CD43 for routine diagnostic purposes, and cytokeratin was used to identify lymphoepithelial lesions [21, 22].

During the similar period (January 2007 and December 2015), patients diagnosed with stage IE1 or stage IIE1 extragastric MALT lymphoma, including OAML, pulmonary, and salivary gland MALT lymphoma, who received first‐line conventional treatment, including radiotherapy, chemotherapy, or rituximab‐based regimen and did not receive first‐line antibiotic treatment, were selected as the control cohort. Patients who received regular follow‐ups and did not receive further treatment, including antibiotics and conventional treatment, after a confirmed diagnosis of extragastric MALT lymphoma, were excluded from this study.

The Research Ethics Committee of the National Taiwan University Hospital approved this study (approval number: 201505013RIND), and written informed consent was obtained from all patients who received first‐line antibiotic treatment, according to the tenets of the Declaration of Helsinki. The medical data of the patients who received conventional treatment in this study were anonymized prior to access and analysis.

### 2.2 Staging processes, antibiotic treatment regimens, and responsiveness assessment

The staging workups included a physical examination with an inspection of Waldeyer's ring; a detailed history; a hemogram with leukocyte differential count; serum lactate dehydrogenase evaluation; CT scan of the head, neck, chest, abdomen, and pelvis; bone marrow aspiration and biopsy; and assessment of hepatitis B virus (HBV) and hepatitis C virus (HCV) infection status [22, 23, 24]. All patients also underwent the assessment of serum beta‐2‐microglobulin, immunoglobulin (Ig) G, IgM, IgA, and serum immunofixation electrophoresis (IFE) before the treatment according to the staging recommendation of the European Gastro‐Intestinal Lymphoma Study consensus report [24, 25].

Stage IE or IIE1 disease was based on an adaptation of the Ann Arbor staging system modified by Musshoff for primary extranodal lymphomas [26]. In this study, stage IE1 was defined as lymphoma confined to the involved site without lymph node involvement, and stage IIE1 was defined as localized involvement of one or more involved site(s) on one side of the diaphragm with regional lymph node involvement. As bilateral ocular adnexal lesions of lymphoma and adjacent lymphatic involvement can be treated by local irradiation, localized OAML was defined in this study as a lymphoma involving unilateral ocular adnexal (stage IE) or bilateral ocular adnexal lesions with adjacent lymphatics (stage IIE1) [27].

Few patients with OAML were initially administered doxycycline for 2 weeks; however, most patients did not respond to this treatment at the follow‐up 1 year after completing doxycycline. The patients were then administered clarithromycin or HPE regimens consisting of 500 mg amoxicillin four times daily or 500 mg clarithromycin twice daily and PPIs comprising 20 mg omeprazole or 30 mg lansoprazole, twice daily for 2 weeks. If the patient was allergic to amoxicillin, it was replaced with 250 mg metronidazole four times daily combined with clarithromycin and PPIs [21, 22].

The present study assessed the treatment response according to the primary lesions of extragastric MALT lymphoma, which were divided into two subgroups: conjunctival and non‐conjunctival lesions. Patients with conjunctival lesions underwent regular local and physical ophthalmologic examinations every 2–3 months after starting treatment, while patients with non‐conjunctival lesions underwent follow‐up CT or MRI examinations every 3 months after starting treatment. For conjunctival lesions of OAML, CR was defined as the complete absence of any clinical evidence of lymphoma on slit‐lamp examination for at least 6 weeks, whereas PR was defined as a disease reduction of at least 50% for 6 weeks [28]. For non‐conjunctival lesions of extragastric MALT lymphomas, the tumor response was assessed according to the 2017 International Working Group consensus response evaluation criteria involving lymphomas [29], in which CR was defined as the complete absence of all lymphoma lesions (if lymph nodes; long axis <10 mm), while a PR was defined as a 30% decrease in the sum of the longest diameters of the lymphoma lesions (including lymph nodes).

### 2.3 Statistical analyses

This study used Fisher's exact tests to compare clinical characteristics, presence of elevated levels of serum IgG, IgA, or IgM, or monoclonal gammopathy, and presence of HP infections between the antibiotic‐responsive (CR or PR) and antibiotic‐unresponsive (stable disease [SD] and progressive disease [PD]) groups after antibiotic treatment. The analyses were conducted using follow‐up data available on December 31, 2020. Event‐free survival (EFS) after first‐line treatment was calculated from the date of initial treatment until treatment failure, including disease progression, relapse, or treatment discontinuation for any reason, including death [30]. Overall survival (OS) was calculated from the date of initial treatment to the date of death from any cause [30]. Statistical significance was set at *p* < 0.05.

## 3 RESULTS

### 3.1 Clinicopathological features and tumor responses to antibiotic treatment

Between January 2010 and December 2015, we informed the patients of the pros and cons of antibiotic treatment as an alternative first‐line therapy for stage IE and IIE1 extragastric MALT lymphoma. In this exploratory study, we retrospectively reviewed 28 patients with extragastric MALT lymphoma who received first‐line antibiotic treatment, including 17 patients with OAML (nine conjunctival MALT lymphoma and eight orbital MALT lymphoma); four patients with salivary gland MALT lymphoma; three patients with pulmonary MALT lymphoma; and one patient each with colon, thyroid, laryngeal, and tracheal MALT lymphomas (Table 1).

**TABLE 1:** Demographics of patients with localized extragastric MALT lymphoma who received first‐line antibiotics treatment

**Characteristics**	**Absolute numbers (percentage)**
**Gender**	Total number: 28
Women	17 (60.7%)
Men	11 (39.3%)
**Median follow‐up in months (95% confidence interval)**	7.0 (6.84–8.36)
**Median age in years (range)**	59.4 (15–84)
**Stage (Ann‐Arbor)**	
IE1	19 (67.9%)
IIE1	9 (32.1%)
**Lesion sites**	
Conjunctiva	9 (32.1%)
Orbit (lacrimal sac)	8 (28.5%)
Parotid gland	4 (14.3%)
Lung	3 (10.7%)
Colon	1 (3.6%)
Thyroid	1 (3.6%)
Larynx	1 (3.6%)
Trachea	1 (3.6%)
**Serum IgG, IgM, or IgA**	
Normal	17 (60.7%)
Elevation of IgG and IgM	3 (10.7%)
Elevation of IgG and IgA	2 (7.1%)
Elevation of IgG	3 (10.7%)
Elevation of IgM	2 (7.1%)
Elevation of IgA	1 (3.7%)
**Serum immunofixation electrophoresis**	
No gammopathy	20 (71.4%)
IgG/κ monoclonal gammopathy	4 (14.3%)
IgG/λ monoclonal gammopathy	3 (10.7%)
IgM/κ monoclonal gammopathy	1 (3.6%)
**Concomitant chronic infections**	
*Helicobacter pylori*	5 (17.9%)
HBV + *H. pylori*	1 (3.6%)
HCV + *H. pylori*	1 (3.6%)
HBV	3 (10.7%)
HCV	1 (3.6%)
**First‐line antibiotics treatment** ^a^	
Doxycycline	2 (7.1%)
Doxycycline (SD), shift to HPE regimen	5 (17.9%)
HPE regimen	19 (67.9%)
Clarithromycin	2 (7.1%)

Abbreviations: HPE, *Helicobacter pylori* eradication; MALT, mucosa‐associated lymphoid tissue; SD, stable disease.

^a^ *Helicobacter pylori* eradication, such as proton pump inhibitors plus amoxicillin, clarithromycin, or metronidazole.

This study included 17 women and 11 men, with a median age of 59.5 years (range: 15–84 years). The initial diagnoses and stages for all patients were as follows: 19 patients had stage IE and nine patients had stage IIE. Seventeen (60.7%) patients had normal IgG, IgM, and IgA levels, whereas 11 patients had elevated IgG, IgM, or IgA, or combined levels. In total, eight patients had monoclonal gammopathy based on the serum IFE assessment (Table 1). Concomitant HBV and HCV infections were found in four and two patients, respectively. Among the 15 patients who underwent assessment for HP infection, seven were diagnosed with gastric HP infection. Among the 28 patients with extragastric MALT lymphoma, four patients had autoimmune disease, including three with Sjogren's syndrome and one with Hashimoto's thyroiditis. Of the 28 patients who received first‐line antibiotic treatment, 19 (67.9%) received HPE regimens; five patients received doxycycline followed by HPE because they failed to respond to doxycycline (PD or SD 1 year after completing doxycycline), two patients received doxycycline alone, and two patients received clarithromycin alone (Table 1).

**FIGURE 1:**
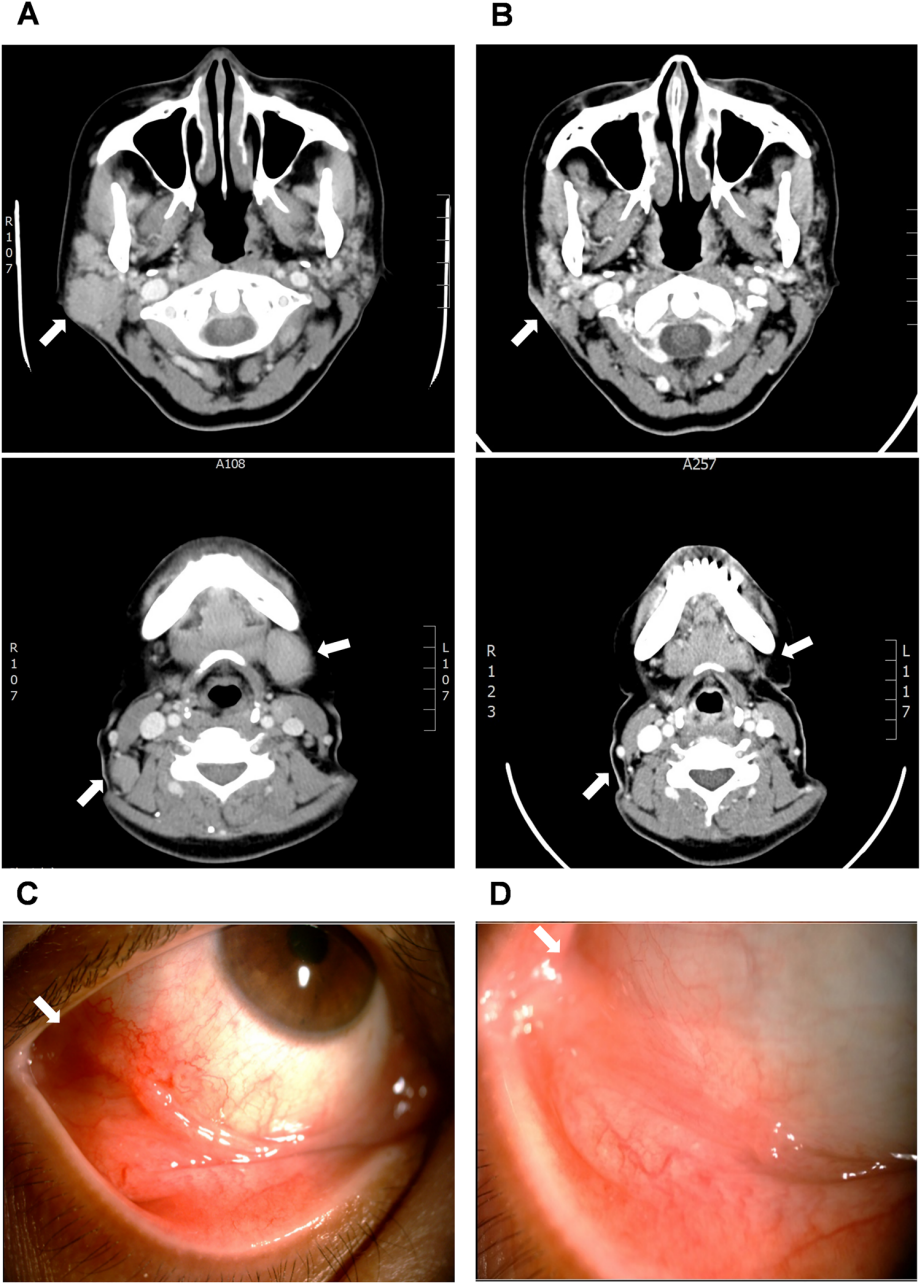
Examples of imaging findings in antibiotic‐responsive cases of extragastric mucosa‐associated lymphoid tissue (MALT) lymphoma after completion of the antibiotic treatment. (A) Case 23, a stage IIE1 parotid gland MALT lymphoma (white arrow; immunophenotypes showed CD20^+^, CD5^−^, CD10^−^, cyclinD1^−^, and some scatted CD3^+^ T cells, CD8^+^, CD4^−^). The patient received a *Helicobacter pylori* eradication (HPE) regimen comprising 1000 mg amoxicillin, 500 mg clarithromycin, and 20 mg omeprazole twice daily for 2 weeks. (B) The patient achieved a complete response (CR) (regression of the right parotid gland tumor and left submandibular lymph nodes, white arrow) 4 months after completing HPE. (C) A 60‐year‐old woman (case 28) with right conjunctival MALT lymphoma (white arrow; CD20^+^, CD5^−^, CD10^−^, CD43^+^, and CD3^−^). The patient received an HPE (30 mg lansoprazole, 500 mg clarithromycin, and 500 mg metronidazole twice daily for 2 weeks). (D) The patient achieved a CR (white arrow) at 5 months after completing HPE; endoscopy showing HP‐positive active gastritis

**FIGURE 2:**
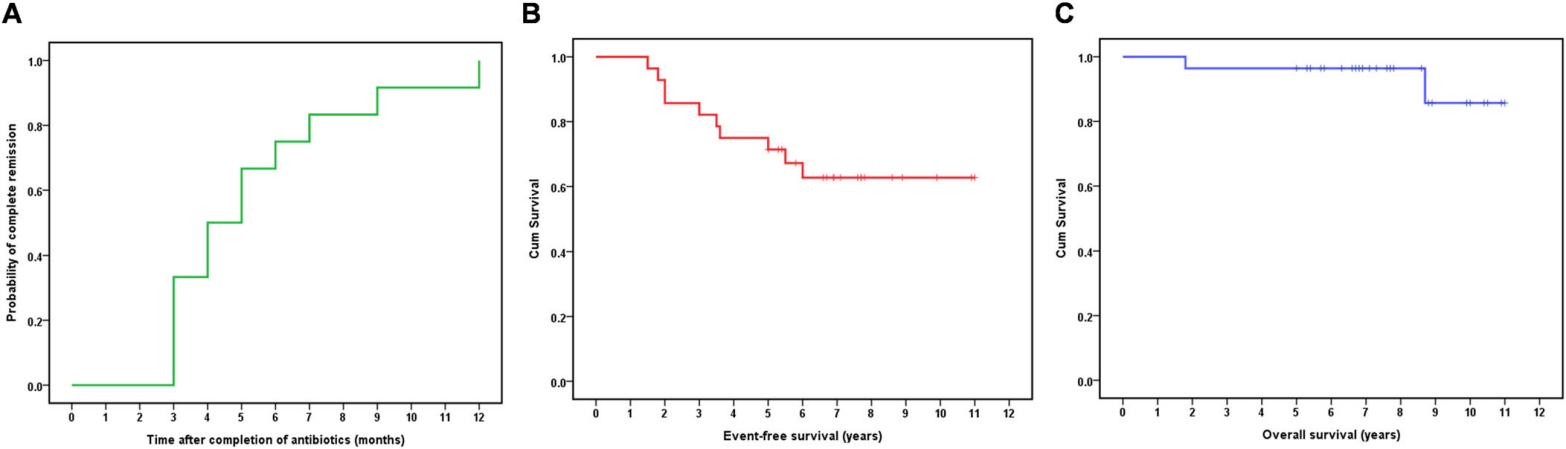
Time to complete remission and survival curves of patients who received antibiotics as a first‐line treatment. (A) The time to complete remission (CR) in 12 cases was calculated from the completion of antibiotic treatment to the first evidence of CR through Kaplan–Meier analysis. (B) Event‐free survival for all cases. (C) Overall survival curve for all cases

Among the patients who received first‐line antibiotic treatment, 12 achieved CR and four achieved PR, whereas 10 and two patients had SD and PD, respectively (Table 2). The ORR for all patients was 57.1% (95% confidence interval [CI]: 37.6%–76.7%). Among the 12 patients with CR, two received doxycycline, two received HPE following doxycycline, and eight patients received HPE. Among the four patients with PR, two received HPE, one received clarithromycin, and one received doxycycline followed by HPE (Table 2). Regarding the association between antibiotic responsiveness (CR and PR) and primary target lesions, we found that 11 patients with OAML, three with salivary gland MALT lymphoma, one patient with colon MALT lymphoma, and one patient with pulmonary MALT lymphoma had antibiotic‐responsive tumors (Figure 1). The median time to CR (*n* = 12) was 4.0 months (range: 3–12 months; 95% CI: 2.3–5.7 months) (Figure 2A).

**TABLE 2:** Tumor locations, antibiotics regimens, treatment responses, parameters, and salvage treatments of patients with extragastric MALT lymphoma

**Case**	**Sex**	**Age**	**Primary lesion**	**Stage**	**Time to CR**	**Response**	**Antibiotics**	**Alive**	**OS (years)**	**TTP (years)**	**HP**	**HBV/HCV**	**Serum Igs**	**Gammopathy**	**Autoimmune disease**	**2nd or 3rd Tx treatment**
1	M	67	Conjunctiva (Rt)	IE1	7.0	CR	Dox	Yes	11.2		NA	Neg	Normal	None	No	
2	F	33	Conjunctiva (Lt)	IE1	4.0	CR	Dox	Yes	10.9		NA	Neg	Normal	None	No	
3	M	76	Conjunctiva (Rt)	IE1		SD	Dox‐SD, HPE	Yes	10.4	6.0	Neg	Neg	Normal	None	No	LP
4	M	66	Orbit (Rt)	IE1	5.0	CR	Dox‐SD, HPE	Yes	6.9		NA	Neg	Normal	None	No	Follow‐up
5	M	78	Orbit (Lt)	IE1		PD	Dox‐PD, HPE	Yes	9.9		NA	Neg	Normal	None	No	Radiotherapy
6	F	66	Conjunctiva (Lt)	IE1	12.0	CR	Dox‐SD, HPE	Yes	6.9		Pos	Neg	Normal	None	No	
7	F	48	Conjunctiva (Bil)	IIE1		PR	Dox‐SD, HPE	Yes	5.3		Neg	Neg	IgM	IgM/kappa	No	Follow‐up
8	M	75	Orbit (Bil)	IIE1		PR	HPE	Yes	5.8		Pos	Neg	Normal	None	No	Follow‐up
9	F	67	Conjunctiva (Rt)	IE1	3.0	CR	HPE	Yes	7.1		NA	HBV	Normal	None	No	
10	F	59	Orbit (Lt)	IE1		PD	HPE	Yes	5.7	1.5	Pos	HBV	Normal	None	No	LP then R‐COP
11	F	58	Lacrimal sac (Rt)	IE1	4.0	CR	HPE	Yes	6.6		Pos	Neg	Normal	None	No	
12	M	33	Conjunctiva (Rt)	IIE1		SD	HPE	Yes	8.8	3.5	NA	HBV	IgG	IgG/lambda	Yes	LP
13	F	84	Trachea	IE1		SD	HPE	Yes	6.3	2.0	NA	Neg	Normal	None	Yes	RP
14	M	72	Larynx	IE1		SD	HPE	Yes	7.3	3.0	Neg	Neg	IgA	None	No	LP
15	F	46	Lung (Bil)	IE1		PR	HPE	Yes	8.9		NA	Neg	IgG & IgA	None	No	Follow‐up
16	F	59	Orbit (Rt)	IE1		SD	HPE	Yes	6.8	5.0	NA	Neg	IgM	IgG/lambda	No	Radiotherapy
17	M	79	Parotid gland (Rt)	IE1		PR	Clarithromycin	Yes	7.8		NA	Neg	Normal	IgG/kappa	No	Follow‐up
18	F	63	Conjunctiva (Bil)	IIE1	9.0	CR	HPE	Yes	8.6		NA	HBV	IgG & IgM	None	No	
19	F	54	Thyroid gland (Bil)	IIE1		SD	HPE	Yes	6.7		NA	Neg	IgG & IgM	IgG/kappa	No	CML^a^
20	F	62	Parotid gland (Bil)	IE1		SD	Clarithromycin	Yes	10.0	5.0	Neg	Neg	IgG & IgA	IgG/lambda	Yes	LP
21	F	50	Lung	IE1		SD	HPE	Yes	7.7		Neg	HCV	Normal	None	No	Follow‐up
22	M	79	Colon	IE1	3.0	CR	HPE	Yes	1.8		Pos	HCV	Normal	None	No	
23	F	52	Parotid gland (Bil)	IIE1	3.0	CR	HPE	Yes	7.7		Neg	Neg	IgG & IgM	None	Yes	
24	M	41	Orbit (Rt)	IE1	6.0	CR	HPE	Yes	7.6		Pos	Neg	Normal	None	No	
25	F	60	Orbit (Bil)	IIE1		SD	HPE	Yes	11.0	3.6	Neg	Neg	IgG	IgG/kappa	No	R‐COP
26	M	15	Parotid gland (Rt)	IIE1	3.0	CR	HPE	Yes	5.0		NA	Neg	Normal	None	No	
27	F	54	Lung (Bil)	IIE1		SD	HPE	Died	8.7	2.0	Neg	Neg	IgG	IgG/kappa	No	R‐COP
28	F	59	Conjunctiva (Rt)	IE1	5.0	CR	HPE	Yes	5.4		Pos	Neg	Normal	None	No	

Abbreviations: Bil, bilateral; CML, chronic myeloid leukemia; CR, complete remission; HP, *Helicobacter pylori*; HPE, HP eradication therapy; Igs, immunoglobulins; LP, chlorambucil and prednisolone; Lt, left; MALT, mucosa‐associated lymphoid tissue; NA, non‐analysis; Neg, negative; OS, overall survival; PD, progressive disease; Pos, positive; PR, partial remission; R‐COP, rituximab plus cyclophosphamide, vincristine, and prednisolone; RP, rituximab and prednisolone; Rt, right; SD, stable disease; TTP, time to progression; Tx, treatment.

^a^ Secondary cancer: treated as CML.

### 3.2 Correlations between clinicopathological features and tumor responses to antibiotic treatment and clinical outcomes, and salvage treatment for antibiotic‐unresponsive tumors

The antibiotic‐responsive rate for 19 patients with stage IE disease was 57.9% (11/19), compared with 55.6% (5/9) in nine patients with stage IIE1 disease (*p* = 0.907). In our study, seven patients with monoclonal gammopathy also had elevated levels of one or all three immunoglobulins (Igs) (IgG, IgA, or IgM), one with monoclonal gammopathy had normal levels of Igs, and four with elevated levels of IgG, IgA, or IgM had an absence of monoclonal gammopathy (Table 2). Elevated levels of one or all three Igs (IgG, IgA, and IgM) were detected in seven (58.3%) of the 12 antibiotic‐unresponsive tumors and four (25%) of the 16 antibiotic‐responsive tumors (*p* = 0.121) (Table 2). Furthermore, immunoglobulin monoclonal gammopathy was significantly correlated with antibiotic‐unresponsive tumors compared to antibiotic‐responsive tumors (50.0% [6/12] vs. 12.5% [2/16], *p* = 0.044). The 15 patients who underwent HP assessment before treatment showed a significant association between HP infection and antibiotic responsiveness (75.0% [6/8] vs. 14.3% [1/7], *p* = 0.041).

At a median follow‐up of 7 years (95% CI: 6.84–8.36 years; range: 1.8–11 years), all patients who achieved CR after antibiotic treatment survived and were lymphoma‐ and progression‐free, whereas four patients who achieved PR received regular follow‐up without further treatment. Detailed secondary and salvage treatment regimens for patients without CR or PR are listed in Table 2. Among the patients who received second‐line chemotherapy, three with chlorambucil and prednisolone, and two with rituximab plus cyclophosphamide, vincristine, and prednisolone, achieved CR, while two patients achieved CR after undergoing radiotherapy. The 7‐year EFS and OS rates for all patients were 62.7% (95% CI: 44.1%–81.3%) and 96.4% (89.5%–100%), respectively (Figure 2B,C).

### 3.3 Clinicopathological features, tumor responses, and outcomes for patients with extragastric MALT lymphoma who received conventional treatment

Between January 2007 and December 2015, 34 men and 30 women with a median age of 57.5 years (range: 25–87 years) were retrospectively analyzed and included in this study. Among them, 31 (48.4%) patients had OAML, 15 (23.5%) had pulmonary MALT lymphoma, and 18 (28.1%) had salivary gland MALT lymphoma. The demographics of the patients who received first‐line conventional treatments, including radiotherapy, chemotherapy, or rituximab‐based regimens, are listed in Table 3. Forty‐one patients had stage IE disease and 23 had stage IIE1 disease (Table 3). Among these patients, 44 received chemotherapy or rituximab‐based regimen (21 OAML, 11 pulmonary MALT lymphoma, and 12 salivary gland MALT lymphoma); and 20 received radiotherapy (10 OAML, four pulmonary MALT lymphoma, and six salivary gland MALT lymphoma). The radiation prescription dose ranged from 30 to 40 Gy (median dose: 32 Gy) in daily fractions of 1.8–2.0 Gy. Chemotherapy or rituximab‐based regimen courses ranging from two to six cycles were used based on the attending physicians’ judgment.

**TABLE 3:** Clinicopathological features and tumor responses in patients with extragastric MALT lymphoma who received conventional treatment

	**Total**	**Ocular adnexal MALToma**	**Pulmonary MALToma**	**Salivary gland MALToma**	***p*‐Value**
Number	64	31	15	18	
Age					0.735
Median (range)	57.5 (25–87)	57.0 (27–87)	59.0 (31–74)	55.5 (25–81)	
Gender					0.654
Women	30 (46.9%)	13 (41.9%)	7 (46.7%)	10 (55.6%)	
Men	34 (53.1%)	18 (58.1%	8 (53.3%)	8 (44.4%)	
Stage					0.264
IE	41 (64.1%)	17 (54.8%)	10 (66.7%)	14 (77.8%)	
IIE	23 (35.9%)	14 (45.2%)	5 (33.3%)	4 (22.2%)	
Response status					0.164
CR +PR	39 (60.9%)	21 (67.7%)	6 (40.0%)	12 (66.7%)	
SD+PD	25 (39.1%)	10 (32.3%)	9 (60.0%)	6 (33.3%)	
Treatment					0.824
Radiotherapy	20 (31.2%)	10 (32.3%)	4 (26.7%)	6 (33.3%)	
Chemotherapy	44 (68.8%)	21 (67.7%)	11 (73.3%)	12 (66.7%)	
Chlorambucil or cyclophosphamide	15 (34.1%)	8 (38.1%)	4 (36.4%)	3 (25.0%)	
COP or CHOP	14 (32.8%)	5 (23.8%)	4 (36.4%)	5 (41.7%)	
Rituximab‐base regimen	15 (34.1%)	8 (38.1%)	3 (27.2%)	4 (33.3%)	

Abbreviations: CHOP, COP plus doxorubicin; COP, cyclophosphamide, vincristine, and prednisolone; CR, complete remission; MALToma, mucosa‐associated lymphoid tissue lymphoma; PD, progressive disease; PR, partial remission; SD, stable disease.

**FIGURE 3:**
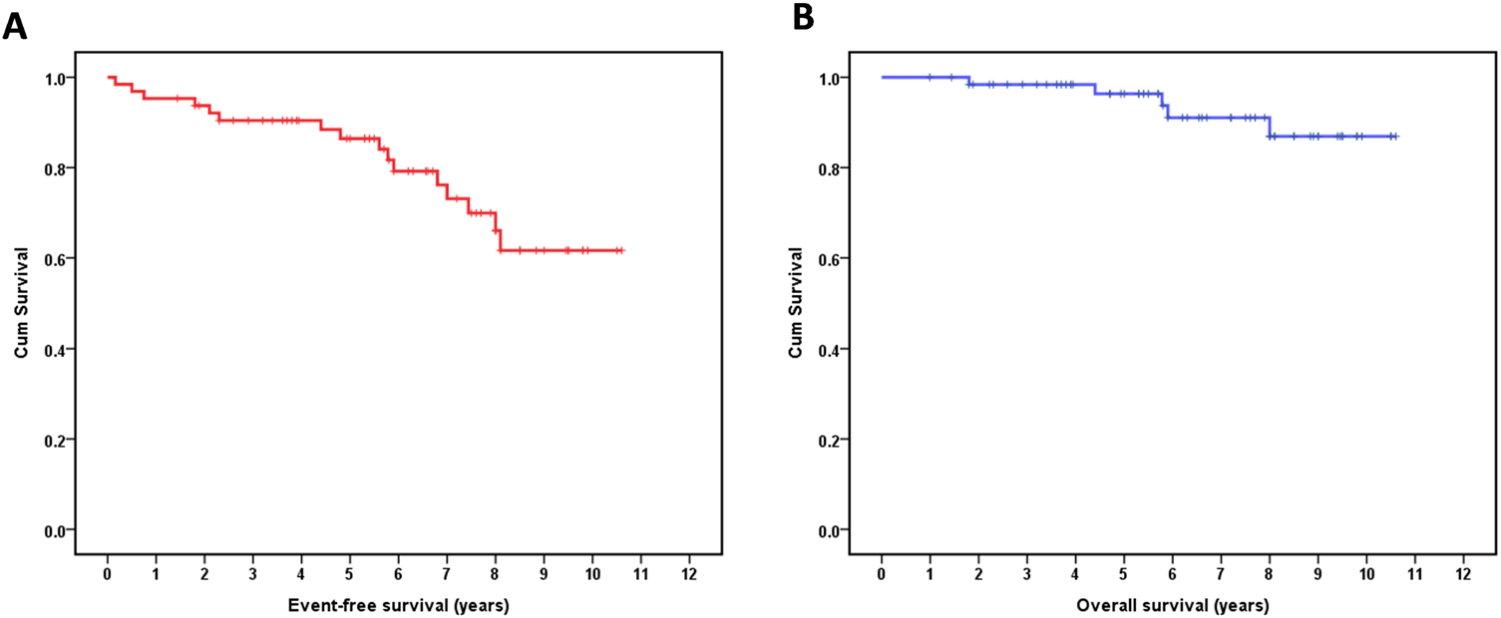
Survival curves of patients who received radiotherapy, chemotherapy, or rituximab‐based regimen as a first‐line treatment. (A) Event‐free survival for all cases. (C) Overall survival curve for all cases

Among the patients with stage IE–IIE1 extragastric MALT lymphoma, the ORR for radiotherapy (*n* = 20) and chemotherapy (including rituximab‐based regimens; *n* = 44) were 70.0% and 56.8%, respectively (*p* = 0.411). During a median follow‐up period of 6.6 years, the 7‐year EFS and OS for this group of tumors were 73.1% (95% CI: 60.0%–86.2%) and 91.1% (95% CI: 82.5%–99.7%), respectively (Figure 3A,B).

As shown in Table S1, no significant differences were observed in the age, sex, stage, ORR, EFS, or OS between patients who received first‐line antibiotic treatment and those who received conventional treatment.

## 4 DISCUSSION

In the current study, we demonstrated that first‐line antibiotics resulted in an ORR of 57.1% in patients with localized extragastric MALT lymphomas. Most importantly, HPE‐like regimens were the main drugs used as first‐line antibiotic treatment in the current study. Furthermore, among patients who underwent assessment for HP infection, the presence of HP infection was correlated with better antibiotic responsiveness. To our knowledge, this is the first report to show that HPE can be used to treat specific group of patients with extragastric MALT lymphoma.

Meanwhile, the clinical response rates (CR, PR, and ORR) and the clinical outcomes (7‐year EFS and OS) of patients who received first‐line antibiotics were not inferior to those of our localized cohort of patients with extragastric MALT lymphoma who received conventional treatment. However, the ORRs and clinical outcomes of our patients who received explorative treatment (first‐line antibiotics) should be cautiously compared with those of similar patients (no difference in age, sex, and stage) who received conventional treatment owing to the selection bias caused by the use of antibiotic treatment, and the heterogeneity of the primary location of extragastric MALT lymphomas may interfere with the presentation of comparisons between these two subgroups [31, 32].

Previous studies have demonstrated that radiotherapy at a conventional dose of 30–40 Gy provides good ORRs and local control rates for patients with localized extragastric MALT lymphomas [4, 5, 33, 34]. In the post hoc analyses of a randomized, phase 3, non‐inferiority trial comparing 24 Gy with 4 Gy for progression‐free survival (PFS) of 84 patients with marginal zone B‐cell lymphoma, Hoskin et al. reported that patients (*n* = 41) receiving 24 Gy had a better 5‐year PFS than those receiving 4 Gy (*n* = 43) (100% vs. 88%, *p* = 0.015) [35].

Despite the excellent clinical response and outcomes after conventional treatment, we advocate the importance of further exploration of antibiotic use as one of the first‐line treatment options for early‐stage extragastric MALT lymphoma based on the following reasons: (a) MALT lymphoma is relatively indolent, and higher grade transformation usually occurs only after long‐term follow‐up if no treatment is administered [36]; (b) antibiotic therapy is well tolerated and associated with excellent quality of life; and (c) conventional treatment can serve as a backbone if patients are not responsive to first‐line antibiotic treatment [3, 4, 37, 38].

In this study, 10 (58.8%) of 17 patients with OAML responded well to first‐line antibiotic treatment, indicating a close association between bacterial infection and OAML tumorigenesis. Although CP infection is associated with OAML oncogenesis in areas with higher incidence [3, 4, 6, 7], corresponding results revealed that the first‐line antibiotics that included doxycycline resulted in an ORR of approximately 48% for OAML. In another study from Austria, Grünberger et al. reported that of the 11 patients with OAML that were treated with doxycycline (200 mg orally daily for 3 weeks), none achieved PR or CR after antibiotic treatment [8], indicating the role of geographic differences in CP in the different treatment efficacies of doxycycline for treating OAML [9, 39–41]. A study by Chanudet et al. investigated the prevalence of CP infection by polymerase chain reaction (PCR) in patients with OAML from six geographic areas, including Germany (47%), the East Coast of the United States (35%), the Netherlands (29%), Italy (13%), the United Kingdom (12%), and Southern China (11%) [39]. Our current findings also showed that two (28.6%) of seven patients receiving first‐line doxycycline achieved CR; however, five patients did not respond to doxycycline. The low prevalence of CP in OAML in East Asian areas, for example, in northern China (0%) and Japan (0%) [42, 43, 44], may explain the lower response rate to doxycycline for OAML in our population.

Although there was no response of OAML to the use of doxycycline for CP eradication, Grünberger et al. reported that four patients had gastric HP infection [8]. Ferreri et al. assessed the presence of HP infection in 31 patients with OAML by endoscopic biopsy followed by histopathologic diagnosis, and reported that 10 (32%) patients had HP infection; however, four of these patients showed no lymphoma regression after receiving HPE as an exclusive treatment [45]. In a study conducted in France, Decaudin et al. reported a 45.9% (28/61) frequency of gastric HP infection in patients with newly diagnosed ocular lymphoma (MALT lymphoma or lymphoplasmacytic lymphoma), which was detected by histology and PCR assay [46]. In the assessment of first‐line doxycycline for the eradication of CP in 47 patients with OAML, Ferreri et al. showed that doxycycline resulted in an ORR of 65% (six with CR and 16 with PR) among 29 patients with CP DNA, with a total of 10 (21.2%) out of 47 patients having gastric HP infection [47]. Our current study also showed that seven (46.7%) of 15 patients who underwent assessment of HP examinations were positive for gastric HP infection. In 2011, Hasosah et al. reported a case of a 6‐year‐old male who presented with localized MALT lymphoma of the lacrimal gland and achieved CR at 12 months after receiving HPE (2 weeks of amoxicillin, clarithromycin, and esomeprazole) and remained lymphoma‐free for 4 years as reported at the final publication date [48]. These findings are supported by those of our current study showing that six (60%) of 10 patients with OAML who received first‐line HPE had CR or PR, and that three patients responded well to HPE after failing to doxycycline.

In addition to patients with OAML, in the current study, three patients with salivary gland MALT lymphoma, one patient with colon MALT lymphoma, and one patient with pulmonary MALT lymphoma responded well to first‐line HPE, indicating that these non‐OAML and extragastric MALT lymphomas may be linked to bacterial infections. Previously, anecdotal case reports showed that two patients with salivary gland MALT lymphoma (a 62‐year‐old woman and a 60‐year‐old man with Sjogren's syndrome presenting with both submandibular and parotid gland involvement, and recurrent parotid gland lesions, respectively) and gastric HP infection achieved CRs of 22 and 6 months, respectively, after receiving first‐line HPE [11, 12]. In an analysis of the efficacy of treatments consisting of amoxicillin and clarithromycin in patients with rectal MALT lymphoma, Niino et al. showed CR in five (62.5%) of eight patients with lymphoma, four (80.0%) of whom had gastric HP infection based on HP test findings [49]. Ishimatsu et al. reported the case of a 57‐year‐old woman presenting with pulmonary MALT lymphoma and HP‐positive atrophic gastritis who subsequently received first‐ and second‐line HPE [10]. The MALT lymphoma lesion achieved CR 6 months after completing second‐line HPE comprising continuous administration of clarithromycin (200 mg/day) [10].

Although the aforementioned case series did not demonstrate the presence of HP infection in tumor samples from the ocular adnexal area, salivary gland, colon, and lung, the possible reasons for using HPE treatment in some patients with extragastric MALT lymphoma with gastric HP infection include the following: (a) B‐lymphocytes located in the gastric microenvironment can be triggered by the continuous stimulation of HP via their interaction with HP‐specific T cells, and gradually transformed into malignant B‐cell clones; these may migrate to extragastric mucosal areas and eventually develop into MALT lymphoma. Thus, HPE eliminates antigen presentation in the stomach and helps HP‐specific T cells (homing to the extragastric microenvironment), to subsequently cause tumor regression and interrupt the lymphocyte homing mechanisms [38, 50, 51]. (b) The organisms involved in HP infection may be associated with the development of some extragastric MALT lymphomas; thus, HP eradication can result in tumor regression, the mechanisms of which are similar to those for the regression of gastric MALT lymphoma by HPE [1, 5, 52]. In a review article on the assessment of HP infection in gastric and extragastric regions, Testerman and Morris suggested that HP may be detected in extragastric microenvironments, such as the eye, nasal cavity, middle ear, oral cavity, coronary artery, skin, liver, peritoneum, large intestine, and gall bladder, based on the assessment of HP culture, PCR methods, and histology methods [53]. (c) We cannot exclude the direct antineoplastic or immunomodulatory effects of antibiotics such as clarithromycin (the main regimen of HPE) [16, 17, 54], which has a reported ORR rate of 52.2% (six with CR and six with PR) in a phase II study exploring the use of 2 g clarithromycin per day (days 1–14, every 21 days) for four courses in 23 patients with relapsed or refractory MALT lymphoma [55].

Based on anecdotal case reports and the results of our current studies showing that HP eradication could result in regression of extragastric MALT lymphoma, it is worth investigating the efficacy of first‐line HPE in the treatment of localized extragastric MALT lymphoma. Our ongoing prospective phase II trial (ClinicalTrials.gov, NCT02987127; started in 2016) is the first study to evaluate the efficacy of the first‐line antibiotic HPE, as determined by the CR rate and time to tumor progression, in patients with localized (stage IE and IIE1) extragastric MALT lymphoma. This trial will also assess the occurrence of HP infection in gastric and extragastric lesions and endeavor to understand whether extragastric MALT lymphoma is responsive to 2 weeks of antibiotic treatment.

In conclusion, the results of the current study demonstrated that first‐line antibiotic treatment, consisting mainly of HPE regimens, resulted in an ORR of 57.1% in patients with localized extragastric MALT lymphoma. The ORR and OS of patients who received first‐line antibiotic treatment did not differ from those of our control cohort who received standard therapy. First‐line antibiotic treatment for these extragastric MALT lymphoma patients not only provided a short course and nontoxic treatment, but also offered an effective treatment strategy. Furthermore, antibiotic treatment can prevent systemic chemotherapy or radiotherapy‐related adverse effects. Our findings support the treatment recommendations for MALT lymphoma proposed by Raderer and Kiesewetter on the use of a first‐line clarithromycin‐based regimen for extragastric MALT lymphoma, particularly OAML [56].

## AUTHOR CONTRIBUTIONS

Ming Yao, Shu‐Lang Liao, and Sung‐Hsin Kuo designed and conducted the study. Ming Yao and Sung‐Hsin Kuo had full access to all of the data in the study, took responsibility for the integrity of the data and the accuracy of the data analysis, and wrote the manuscript. Ming Yao, Shu‐Lang Liao, Cheng‐Ping Wang, Wei‐Li Ma, Yi‐Hsuan Wei, Jyh‐Ming Liou, I‐Jong Wang, Ann‐Lii Cheng, and Sung‐Hsin Kuo treated patients and provided the data. Chung‐Wu Lin reviewed the pathological characteristics and immunohistochemical studies. Sung‐Hsin Kuo edited the manuscript and reviewed the manuscript. All authors had performed critical revision and approved of the manuscript for important intellectual content.

## CONFLICT OF INTEREST

The authors declare that they have no conflict of interest.

## ETHICS STATEMENT

The Research Ethics Committee of the National Taiwan University Hospital approved this study (approval number: 201505013RIND), and written informed consent was obtained from all patients who received first‐line antibiotic treatment, according to the tenets of the Declaration of Helsinki. The medical data of the patients who received conventional treatment in this study were anonymized prior to access and analysis.

## Supporting information

Supporting Information

## Data Availability

The anonymized data obtained and analyzed in this study are available from the corresponding author upon reasonable request.
