# Supplementary material for: First‐line antibiotic treatment in patients with localized extragastric mucosa‐associated lymphoid tissue lymphoma
Source: EJHaem. 2022 Nov 6;4(1):55–66. doi: 10.1002/jha2.608 (PMC9928665; doi:10.1002/jha2.608)
Supplement: Supplementary file 1 — Supporting Information [file JHA2-4-55-s001.docx]

**Supplementary Table 1. Clinicopathological features, tumor responses, and clinical outcomes in patients with extragastric MALT lymphoma who received first-line antibiotics or conventional treatment**

|  | First-line antibiotics treatment | Conventional treatment | *p-*value |
| --- | --- | --- | --- |
| Number | 28 | 64 |  |
| Age |  |  | 0.375 |
| Median (range) | 59.4 (15-84) | 57.5 (25-87) |  |
| Gender |  |  | 0.262 |
| Women | 17 (60.7%) | 30 (46.9%) |  |
| Men | 11 (39.3%) | 34 (53.1%) |  |
| Stage |  |  | 0.814 |
| IE | 19 (67.9%) | 41 (64.1%) |  |
| IIE | 9 (32.1%) | 23 (35.9%) |  |
| Response status |  |  | 0.819 |
| CR + PR | 16 (57.1%) | 39 (60.9%) |  |
| SD + PD | 12 (42.9%) | 25 (39.1%) |  |
| Clinical outcomes |  |  |  |
| 7-year EFS | 62.7% | 73.1% | 0.372 |
| 7-year OS | 96.4% | 91.1% | 0.717 |

Abbreviation: CR, complete remission; PR, partial remission; SD, stable disease; PD, progressive disease; EFS, event-free survival; OS, overall survival.
